# Low back pain-related healthcare utilization following intraosseous basivertebral nerve radiofrequency ablation: a pooled analysis from three prospective clinical trials

**DOI:** 10.1093/pm/pnad114

**Published:** 2023-08-29

**Authors:** Zachary L McCormick, Timothy Curtis, Amanda Cooper, Margo Wheatley, Matthew Smuck

**Affiliations:** Department of Physical Medicine and Rehabilitation, University of Utah School of Medicine, Salt Lake City, UT 84108, United States; Department of Physical Medicine and Rehabilitation, University of Utah School of Medicine, Salt Lake City, UT 84108, United States; Department of Physical Medicine and Rehabilitation, University of Utah School of Medicine, Salt Lake City, UT 84108, United States; Health Economist, Technomics Research, LLC, Medina, MN 55356, United States; Department of Orthopaedic Surgery, Stanford University School of Medicine, Redwood City, CA 94063, United States

**Keywords:** chronic low back pain, basivertebral nerve, radiofrequency ablation, Modic changes, vertebrogenic pain, disc

## Abstract

**Background:**

The effectiveness and safety of intraosseous basivertebral nerve ablation (BVNA) for treating vertebrogenic pain is established, but low back pain-related healthcare utilization (LBPr-HU) following BVNA continues to be defined.

**Methods:**

LBPr-HU data were pooled from 3 prospective studies. LBPr-HU categories of interest included non-invasive conservative care, opioid utilization, lumbosacral spinal injection (LSI), lumbosacral radiofrequency ablation (LRFA), and lumbosacral spinal surgery. Pre- and post-BVNA LBPr-HU were compared at both 1- and 5-years using McNemar’s test for proportions and paired t-tests for means.

**Results:**

Two hundred forty-seven patients received BVNA and had 1-year follow-up; 205 had long-term follow-up (mean of 5.3 ± 1.33 years). Twenty-seven percent fewer participants initiated conservative care in the year post-BVNA compared to the year preceding BVNA (*P* < .001; 95% CI 19.8–34.5). Of 77/247 participants taking opioids at baseline, 40.3% and 61.7% fewer were taking them at one-year and 5.3 ± 1.33 years post-BVNA, respectively (*P* < .001). Of participants receiving LSIs in the year preceding BVNA, 81.2% fewer received LSI(s) in the year post-BVNA (*P* < .001; 95% CI 70.7–90.7); a 76.4% reduction in LSIs was maintained through a mean of 5.3 ± 1.33 years post-BVNA. LRFA rates were 1.6% at 1-year post-BVNA and 8.3% at 5.3 ± 1.33 years post-BVNA. Lumbar fusion surgery was 0.8% at 1-year post-BVNA and 6.5% at 5.3 ± 1.33 years post-BVNA.

**Conclusions:**

In this aggregate analysis of patients with vertebrogenic pain, utilization of conservative care, opioids, LSIs, and LRFA were substantially reduced through 5 years post-BVNA compared to baseline. Lumbar fusion rates were less than half the published value at 5 years in similar populations.

## Background

Chronic low back pain (LBP) is a symptom for a heterogenous group of causative conditions.^1^ Historically, clinicians had limited tools to differentiate between the many potential sources of chronic LBP. This resulted in varied and non-specific treatment approaches with poor effect sizes, and potential for over-treatment.^2–4^ Recent advances in spine biochemistry, biomechanics, epidemiology, and pathophysiology enable a more anatomically-specific approach to the diagnoses and treatment of certain chronic LBP subgroups.^1^

Among various factors, isolating the dominant source of chronic LBP includes a clinical assessment of the pain location (eg, lateral vs midline, radiating vs non-radiating, etc.) and the movements or positions that exacerbate pain to determine the region of pain (eg, anterior vs posterior spinal elements). Imaging and response to diagnostic tests are often used to further isolate the pain source to specific anatomic structures. One structural source of anterior element pain is vertebral endplate pain (“vertebrogenic” pain).^1^^,^^5^

Within the disco-vertebral complex, vertebrogenic pain results from hypersensitized, damaged vertebral endplates that are innervated by the basivertebral nerve (BVN).^6–10^ Vertebral endplates have a higher density of nociceptors than the adjacent intervertebral discs,^9^ and when damaged, molecular interactions occur between the tissues of the endplate/bone marrow complex and the adjacent disc that produce, in some individuals, edema and bone marrow changes with subsequent BVN sensitization.^11^ A presumed diagnosis of vertebrogenic pain is made when a patient’s clinical presentation is consistent with anterior spinal element pain and magnetic resonance imaging (MRI) demonstrates edema and/or fatty infiltrative bone marrow changes at relevant vertebral endplates (Type 1 and/or Type 2 Modic changes).^8^

Radiofrequency ablation (RFA) of the BVN interrupts pain transmission from damaged and hypersensitized vertebral endplates. The effectiveness and safety of intraosseous basivertebral nerve ablation (BVNA) for the treatment of dominant vertebrogenic LBP has been established in two randomized controlled trials (RCTs),^12–14^ 5 prospective single arm cohort studies,^15–20^ and 3 independent meta-analyses. ^21–23^

Reductions in non-surgical treatments, opioid pain medications (“opioids”), and steroid injections as well as low rates of pain and surgical interventions have been reported in study cohorts following BVNA.^24^^,^^25^ In this report, we use pooled clinical outcome data and low back pain-related healthcare utilization (LBPr-HU) data derived from 3 clinical trials to more comprehensively analyze the LBPr-HU of BVNA in patients with dominant vertebrogenic LBP.

## Methods

### Study design

The present study analyzed aggregate clinical outcomes and utilization data from three clinical trials sponsored by Relievant Medsystems, Inc. (Minneapolis, MN, USA). The 3 trials were (1) a prospective, double-blind, randomized sham-control trial (SMART)^12^ with a prospective, open-label, single-arm five-year follow-up study of the BVNA treatment arm^24^; (2) a prospective, open-label RCT comparing BVNA to non-surgical care (INTRACEPT)^13^^,^^14^ with an optional prospective, open-label, single-arm 3-, 4-, and 5-year follow-up study of the BVNA treatment arm; and (3) a prospective, open-label, single-arm cohort study^16^^,^^17^ with an optional 3-, 4-, and 5-year follow-up study of BVNA-treated participants.

Each study was registered on ClinicalTrials.gov: NCT01446419 (SMART RCT), NCT03997825 (SMART 5+ Year Follow-up Study), NCT03246061 (INTRACEPT RCT), NCT03266107 (CLBP Single-Arm Cohort Main Study), and NCT05207813 (CLBP Single-Arm Long-Term Study). The studies were compliant with Health Insurance Portability and Accountability Act (HIPAA), Good Clinical Practices, and the Declaration of Helsinki, and were conducted under Institutional Review Board approval and participant informed consent.

### Study population

All participants enrolled in the 3 original studies had refractory CLBP for a minimum of 6 months, with Modic changes (Type 1 and/or Type 2 from L3 to S1) as the objective imaging biomarker for primary vertebrogenic pain. Baseline MRIs, participant-completed pain body diagrams, and inclusion/exclusion documentation were reviewed by up to 3 independent orthopedic spine medical reviewers to confirm the presence of Type 1 and/or Type 2 Modic changes and the diagnosis of the dominant pain source as vertebrogenic.

Participants in the 3 studies were identified from existing practice populations, physician referrals, and patient self-referrals and were enrolled between October 2011 and February 2019 at 31 study sites in the United States and 3 in Europe. In the 2 RCTS, consecutive patients with suspected vertebrogenic pain were approached and randomized after consent. For the single-arm cohort study, consecutive patients were approached to participate, with all patients being treated with BVNA. Primary inclusion/exclusion criteria, study endpoints, data collection tools, and protocol requirements were similar for the 3 studies, allowing for data pooling.

The primary inclusion and exclusion criteria for the three pooled studies are listed in Table 1. Enrollment criteria allowed for moderate spinal stenosis without associated neurogenic symptoms, previous non-fusion lumbar spine surgeries (eg, discectomies and laminectomies) if > 6 months prior to baseline and no ongoing radicular symptoms, disc extrusions/protrusions ≤ 5 mm, and spondylolisthesis ≤ 2 mm. Compared to the RCT studies, the CLBP prospective single-arm cohort study was more lenient in its enrollment criteria with regard to opioids and body mass index (BMI), allowing for inclusion of patients with extended-release opioid use and BMI > 40 (provided the procedure could be technically completed).

**Table 1.** Inclusion and exclusion criteria.

Inclusion Criteria	Exclusion Criteria
Skeletally mature patients with chronic (≥ 6 months) isolated lumbar back pain, who had not responded to at least 6 months of non-operative management Type 1 or Type 2 Modic changes at one or more vertebral body for levels L3 to S1 Minimum ODI of 30 points (100-point scale) Minimum VAS of 4 cm (10 cm scale) Ability to provide informed consent, read and complete questionnaires	MRI evidence of Modic changes at levels other than L3 to S1 Radicular pain (defined as nerve pain following a dermatomal distribution and that correlates with nerve compression in imaging) Previous lumbar spine surgery (discectomy/laminectomy allowed if > 6 months prior to baseline and radicular pain resolved)^a^ Symptomatic spinal stenosis (defined as the presence of neurogenic claudication and confirmed by imaging) Metabolic bone disease, spine fragility fracture history, trauma/compression fracture, or spinal cancer Spine infection, active systemic infection, bleeding diathesis Radiographic evidence of other low back pain etiology Disc extrusion or protrusion > 5 mm Spondylolisthesis > 2 mm at any level Spondylolysis at any level Facet arthrosis/effusion correlated with facet-mediated LBP Beck Depression Inventory > 24 or 3 or more Waddell’s signs Compensated injury or litigation Currently taking extended-release opioids with addiction behaviors^b^ BMI > 40^b^ Bedbound or neurological condition that prevents early mobility or any medical condition that impairs follow-up^b^ Contraindication to MRI, allergies to components of the device, active implantable devices, pregnant or lactating

Table 1 lists the inclusion and exclusion criteria for the three studies with footnotes for any differences between the individual studies.

Abbreviations: BMI = body mass index; MRI = magnetic resonance imaging; ODI = Oswestry Disability Index; VAS = Visual Analogue Scale (average low back pain in past 7 days).

a Prior discectomy and laminectomy allowed in INTRACEPT and CLBP Single Arm Cohort Study only. SMART excluded all patients with previous lumbar spine surgery.

b Exclusion criteria for the SMART and INTRACEPT trials only.

### Follow-up visits

The original RCT main study protocols required up to 2-years of follow-up, with timepoints at 6 weeks and 3, 6, 12, and 24 months for the SMART study and at 6 weeks and 3, 6, 9, 12, and 24 months for the INTRACEPT study. The CLBP Single Arm Cohort main study had 1 year of follow-up with timepoints at 6 weeks and 3, 6, 9, and 12 months. All 3 studies included common follow-up timepoints at 3, 6, and 12 months. Patient-reported outcomes of Oswestry Disability Index (ODI), Visual Analog Scale (VAS), and SF-36 along with opioid medication use and LBP treatments (non-invasive conservative therapy, lumbosacral spinal injections, lumbosacral spine radiofrequency ablation, spinal cord stimulation, and lumbosacral surgeries) were collected at each study visit through 12 months.

Each study had an optional long-term follow-up study of the BVNA treatment arm participants. Main study participants were approached at their 24 month study visit (INTRACEPT) or by telephone at the 3-year visit timeframe (CLBP Single Arm Cohort Study) or 5-year visit timeframe (SMART). Fourteen participants in Europe were not invited to participate in the long-term study due to difficulties in contacting them. Long-term visits were performed at 3, 4, and 5 years (INTRACEPT and CLBP Single Arm Cohort Study) and 5 years (SMART 5+ Year Follow-up Study) with ODI, VAS, activity level, work status, opioid medication use, lumbosacral injections (LSI), lumbosacral spine radiofrequency ablation (LRFA), and low back surgeries collected post BVNA for all 3 studies. Table 2 outlines the follow-up visits for each study.

**Table 2.** Alignment and follow-up visit data for pooled analysis.

Study	Baseline	2 Weeks	6 Weeks	3 Months	6 Months	9 Months	1 Year	2 Years	3 Years	4 Years	5 Years
SMART	O, U	O	M	O, U	O, U		O, U	O, U^a^			O, U
INTRACEPT	O, U		M	O, U	O, U	O, U	O, U	O, U	O, U	O, U	O, U
Single-Arm Cohort Study	O, U		M	O, U	O, U	O, U	O, U		O, U	O, U	O, U

Table 2 outlines the protocol-required study visits for the main study and the optional long-term follow-up study for the three pooled studies included in this utilization analysis.

Abbreviations: M = Follow-up visit with only magnetic resonance imaging (MRI) and adverse events collected. O = Follow-up visit with clinical outcomes of Oswestry Disability Index and Visual Analog Scale/Numeric Pain Score collected at all outcome visits. U = Follow-up visit with utilization collected, including non-interventional conservative care (through 24 months only), opioid medication utilization, spinal injections, lumbosacral RFA, and lumbosacral spine surgeries.

a SMART utilization data collection included lumbosacral injections, lumbosacral radiofrequency ablation, and lumbosacral spine surgeries. No opioid data were collected at the 24-month visit for SMART.

### Intervention

BVNA was conducted within each vertebral body exhibiting Modic changes (L3 to S1) using the same technique and the Intracept ^®^ System (Relievant Medsystems, Minneapolis, MN USA) in each study. The full procedure has been described previously.^12^^,^^13^^,^^16^ Treatments provided following randomization and post-BVNA were based on a shared decision-making process between the participant and the treating physician, including but not limited to the following: physical therapy, exercise, chiropractic treatment, acupuncture, oral pain medications, LSI, LRFA, and lumbosacral spine surgery. For the purposes of the present study, treatments were categorized for analysis as (1) non-invasive conservative care, (2) opioid medication utilization (“opioids”), (3) diagnostic and therapeutic lumbosacral spinal injection (including epidural, intradiscal, trigger point, medial branch/facet joint, lateral branch/sacroiliac joint injections), (4) lumbosacral radiofrequency ablation (LRFA), and (5) lumbosacral spinal surgery.

### Utilization data collection

All 3 original main studies used medication and treatment logs, as well as patient interviews, to collect historical LBP-related treatment utilization data from onset of LBP up to the point of baseline and at each study visit post-BVNA. Pre-baseline data were monitored to demonstrate a minimum of 6 months of conservative care and may not be inclusive of all treatments prior to enrollment. As such the utilization pre-baseline may be underestimated. Additionally, any LBP treatments reported for an adverse event (AE) were collected on the AE case report form. Medical records, medication logs, and treatment logs were reviewed by independent study monitors to ensure comprehensive reporting. In the optional long-term follow-up studies, patient-reported outcomes and LBP treatments were collected via interview by the study site clinical research coordinator (INTRACEPT long-term follow-up study) or an independent clinical research associate (SMART 5+ and CLBP single-arm long-term follow-up studies). Study data were aggregated into a single validated clinical study database (Clindex version 5 software; Fortress Medical, Minneapolis, MN, USA).

All post-BVNA LSIs (including epidural, intradiscal, trigger point, medial branch/facet joint, lateral branch/sacroiliac joint injections), pain interventions, and surgeries indicated for the treatment of LBP were adjudicated by an independent Clinical Event Committee (CEC) comprised of 2 orthopedic spine surgeons, who determined whether the involved level(s) and pain source(s) were the same for the additional treatment as when BVNA was performed. The pain source was conservatively deemed the “same” dominant vertebrogenic pain source if there was no response to the additional treatment and/or the pain source could not be anatomically isolated beyond discovertebral disease.

### Statistical analyses

To evaluate if major differences existed between studies prior to aggregation, baseline demographic and clinical characteristics as well as healthcare utilization at any time prior to baseline were analyzed and compared across individual studies using Fisher’s exact test. Healthcare utilization was compared 1-year pre-baseline vs 1-year post-BVNA for an aggregate cohort of 247 participants who completed a 1-year follow-up visit. Long-term healthcare utilization was compared 5-years pre-baseline vs any time post-BVNA for an aggregate cohort of 205 participants who completed at least one long-term follow-up visit at year(s) 3, 4, or 5 (mean follow-up length of 5.3 years). Comparisons for pre-post analyses were conducted using McNemar’s test for proportions and paired t-tests for means using a 0.05 significance level. Kaplan-Meier curves were generated to evaluate time to first LBP-related pain intervention/surgery following BVNA treatment. Data were analyzed as observed with no imputations for missing data. Statistical analyses were performed with SAS version 9.4 software (SAS Institute, Cary, NC, USA) and STATA version 17.0 software (StataCorp LLC, College Station, TX, USA).

## Results

### Study participant disposition

**Figure 1.**
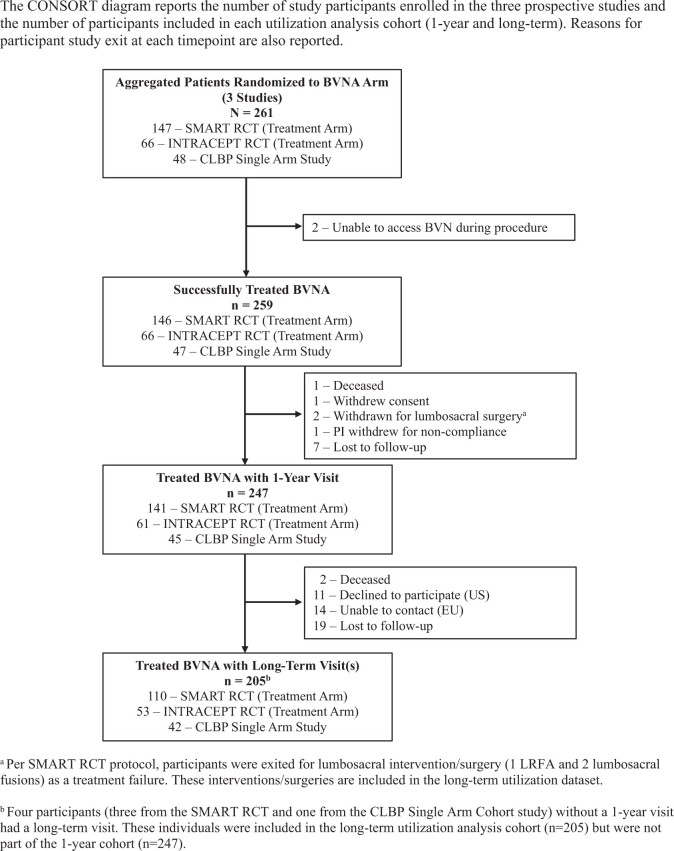
CONSORT diagram of the aggregate cohort included in the analysis.

A combined total of 261 study participants with presumed vertebrogenic pain (confirmed by a combination of clinical inclusion criteria and presence of Modic Type 1 and/or Type 2 changes on MRI) were randomized to the BVNA arm in the pooled main studies SMART RCT (*N* = 147), the INTRACEPT RCT (*N* = 66), and the prospective single-arm cohort study (*N* = 48). Treatment was unsuccessful for 2 participants whose basivertebral nerve could not be accessed during the procedure due to hardened bone. Of the remaining 259 BVNA arm study participants, 247 (95%) had a one-year follow-up visit and comprise the cohort for the main study utilization results. Of the 259 BVNA participants with at least 1 long-term follow-up visit, 205 (79%) agreed to participate and comprise the long-term cohort in this analysis. Details on study participant disposition are reported in Figure 1.

### Baseline clinical characteristics

Baseline characteristics for participants in the aggregate cohort (*N* = 247 BVNA treatment arm participants with a one-year study visit) are reported in Table 3 for both the pooled and individual studies. The mean age was 47.3 years (range 25–69 years); 46.2% were female, 66.8% had LBP for ≥ 5 years, and 31.2% were taking opioids at baseline. One or more therapeutic LSI(s) was performed anytime preceding baseline in 59.1% of participants, 8.1% were previously treated with LRFA of the facet joint(s), and 5.3% had prior non-fusion lumbosacral surgery (discectomy/microdiscectomy or laminectomy/laminotomy) preceding baseline. Study participants reported severe pain and disability at baseline with a mean VAS of 6.8 ± 1.3 and mean ODI of 44.0 ± 10.8. Mild to moderate levels of anxiety and/or depression were reported by 33.6% of study participants (that met the exclusion threshold of 30 for Beck Depression Index [BDI]). Participants reported a low Quality of Life (QoL) at baseline with mean scores of 32.9 ± 7.0 and 52.8 ± 10.5 for SF-36 physical component score (PCS) and SF-36 mental component score (MCS), respectively.

**Table 3.** Baseline clinical characteristics by Aggregate Cohort and Individual Study.

Baseline Characteristics	SMART BVNA Arm (N = 141)^a^	INTRACEPT BVNA Arm (*N *= 61)^a^	CLBP Single Arm Cohort BVNA Arm (*N* = 45)^a^	*P* value^b^	BVNA Aggregate Cohort (*N* = 247)^a^
Age in years, mean (range)	47.2 (26–69)	49.8 (30–68)	44.2 (25–66)	**.018**	47.3 (25–69)
Female, n (%)	61 (43.3%)	29 (47.5%)	24 (53.3%)	.501	114 (46.2%)
Duration LBP ≥ 5 years, n (%)	93 (66.0%)	40 (65.6%)	32 (71.1%)	.821	165 (66.8%)
Pfirrmann grade, n (%)					
Pfirrmann grade <3	2 (1.4%)	0 (0.0%)	1 (2.2%)	.573	3 (1.2%)
Pfirrmann grade 3	27 (19.1%)	11 (18.0%)	11 (24.4%)	.688	49 (19.8%)
Pfirrmann grade 4	58 (41.1%)	27 (44.3%)	19 (42.2%)	.907	104 (42.1%)
Pfirrmann grade 5	54 (38.3%)	23 (37.7%)	14 (31.1%)	.676	91 (36.8%)
Foraminal stenosis, n (%)	93 (66.0%)	46 (75.4%)	26 (57.8%)	.156	165 (66.8%)
Subarticular zone stenosis, n (%)	1 (0.7%)	2 (3.3%)	1 (2.2%)	.277	4 (1.6%)
Central canal stenosis, n (%)	6 (4.3%)	5 (8.2%)	2 (4.4%)	.531	13 (5.3%)
Disc protrusion < 5 mm, n (%)	20 (14.2%)	16 (26.2%)	12 (26.7%)	.051	48 (19.4%)
Olisthesis (< grade 1), n (%)	10 (7.1%)	8 (13.1%)	0 (0.0%)	.029	18 (7.3%)
History of non-fusion lumbosacral surgery, n (%)	0 (0.0%)	7 (11.5%)	6 (13.3%)	**<.001**	13 (5.3%)
Mean ODI, mean, SD, (range)	42.9, 10.8, (30.0, 76.0)	44.3, 11.1, (30.0, 76.0)	47.0, 9.9, (30.0, 72.0)	.082	44.0, 10.8, (30.0, 76.0)
Mean VAS, mean, SD, (range)	6.8, 1.4, (4.0, 10.0)	6.6, 1.3, (4.0, 9.0)	6.8, 1.0, (4.0, 9.2)	.732	6.8, 1.3, (4.0, 10.0)
Mean SF-36 (PCS), mean, SD, (range)	33.4, 7.2, (14.8, 48.1)	32.1, 6.5, (18.4–46.1)	32.2, 6.9, (18.6, 48.0)	.366	32.9, 7.0, (14.8, 48.1)
Mean SF-36 (MCS), mean, SD, (range)	52.0, 11.1, (23.1, 69.1)	53.5, 8.8, (33.2, 69.8)	54.2, 10.8, (19.8, 68.0)	.387	52.8, 10.5, (19.8, 69.8)
Mean Beck Depression Index, mean, SD, (range)	7.6, 5.7, (0.0, 23.0)	6.4, 5.3, (0.0, 20.0)	4.5, 3.6, (0.0, 13.0)	**.003**	6.8, 5.4, (0.0, 23.0)
Reported anxiety/depression, mean, SD, (range)	50 (35.5%)	18 (29.5%)	15 (33.3%)	.700	83 (33.6%)

In Table 3, demographic features and clinical characteristics at baseline for BVNA arm aggregate study participants (*N*=247 with a 1-year study visit) are reported for the pooled and individual study results.

Abbreviations: BVNA = basivertebral nerve ablation; LBP = low back pain; MCS = mental component score; ODI = Oswestry disability index; PCS = physical component score; SD = standard deviation; SF-36 = 36-item short form survey; VAS = visual analog scale.

a BVNA arm cohort with a successful procedure and a 1-year visit.

b *P* values using Fisher’s Exact test for categorical data and independent sample t-test for continuous data.

Significant differences are noted in participant age and the BDI scores between the 3 study populations. Prior predictive analyses showed baseline BDI to be a weak predictor of treatment failure^26^ potentially resulting in higher utilization; therefore, no adjustments were made for pooling of the populations. Table 3 reports baseline characteristics.

### Baseline prior low back pain treatments

LBP treatment history including LSI(s), LRFA(s), and lumbar surgeries prior to baseline and opioid use at baseline for BVNA arm aggregate study participants (*N* = 247 with a 1-year study visit), are reported by number and percent of participants for the pooled and individual studies. The rate of conservative treatment is reflective of the requirement that all participants have a minimum of six months of failed conservative treatment to be eligible for the studies (in alignment with the indication for BVNA). The SMART trial had a higher rate of physical therapy in the 12 months preceding baseline, while the other studies had higher rates of intradiscal injections, LRFA, and lumbosacral surgery.

There were significant differences between the 3 studies in the proportion of participants with conservative treatments, the number of intradiscal injections, and prior lumbosacral surgeries. Prior pain interventions and surgery suggests greater comorbidity and a propensity for higher utilization. Therefore, no adjustments were made for pooling of the populations in this analysis on utilization post-BVNA. Table 4 reports low back treatments prior to baseline.

**Table 4.** Low back pain treatments prior to baseline by Aggregate Cohort and Individual Study.

Treatment Type	SMART BVNA Arm (N = 141)^a^	INTRACEPT BVNA Arm (N = 61)^a^	CLBP Single Arm Cohort BVNA Arm (N = 45)^a^	*P* value^b^	BVNA Aggregate Cohort (*N* = 247)^a^
Conservative care prior to baseline [n (%)]	141 (100%)	55 (90.2%)	42 (93.3%)	**<.001**	238 (96.4%)
Acupuncture	28 (19.9%)	10 (16.4%)	3 (6.7%)	.103	41 (16.6%)
Chiropractic care	89 (63.1%)	31 (50.8%)	25 (55.6%)	.242	145 (58.7%)
Physical therapy	141 (100%)	45 (73.8%)	34 (75.6%)	**<.001**	220 (89.1%)
Opioid use at baseline [n (%)]	48 (34.0%)	21 (34.4%)	8 (17.8%)	.098	77 (31.2%)
Diagnostic LSIs prior to baseline [n (%)]	2 (1.4%)	3 (4.9%)	0 (0.0%)	.176	5 (2.0%)
Epidural	0 (0.0%)	0 (0.0%)	0 (0.0%)	1.000	0 (0.0%)
Intradiscal	0 (0.0%)	0 (0.0%)	0 (0.0%)	1.000	0 (0.0%)
Facet joint	2 (1.4%)	3 (4.9%)	0 (0.0%)	.176	5 (2.0%)
Trigger point	0 (0.0%)	0 (0.0%)	0 (0.0%)	1.000	0 (0.0%)
Other	0 (0.0%)	0 (0.0%)	0 (0.0%)	1.000	0 (0.0%)
Therapeutic LSIs prior to baseline [n (%)]	83 (58.9%)	38 (62.3%)	25 (55.6%)	.781	146 (59.1%)
Epidural	72 (51.1%)	31 (50.8%)	22 (48.9%)	.984	125 (50.6%)
Intradiscal	0 (0.0%)	6 (9.8%)	0 (0.0%)	**<.001**	6 (2.4%)
Facet joint	26 (18.4%)	10 (16.4%)	4 (8.9%)	.328	40 (16.2%)
Nerve root	3 (2.1%)	1 (1.6%)	3 (6.7%)	.233	7 (2.8%)
SI joint	1 (0.7%)	1 (1.6%)	0 (0.0%)	.675	2 (0.8%)
Trigger point	2 (1.4%)	2 (3.3%)	0 (0.0%)	.501	4 (1.6%)
Other	1 (0.7%)	1 (1.6%)	1 (2.2%)	.394	3 (1.2%)
LRFA prior to baseline [n (%)]	7 (5.0%)	10 (16.4%)	3 (6.7%)	**.029**	20 (8.1%)
Medial branch	7 (5.0%)	10 (16.4%)	3 (6.7%)	**.029**	20 (8.1%)
Lateral branch	0 (0.0%)	0 (0.0%)	0 (0.0%)	1.000	0 (0.0%)
Basivertebral nerve	0 (0.0%)	0 (0.0%)	0 (0.0%)	1.000	0 (0.0%)
Prior lumbosacral surgery (discectomy/laminectomy) [n (%)]^c^	0 (0.0%)	7 (11.5%)	6 (13.3%)	**<.001**	13 (5.3%)
Discectomy/microdiscectomy	0 (0.0%)	3 (4.9%)	4 (8.9%)	**.001**	7 (2.8%)
Laminectomy/laminotomy	0 (0.0%)	4 (6.6%)	2 (4.4%)	**.006**	6 (2.4%)
Total disc replacement	0 (0.0%)	0 (0.0%)	0 (0.0%)	1.000	0 (0.0%)
Fusion	0 (0.0%)	0 (0.0%)	0 (0.0%)	1.000	0 (0.0%)
Neurostimulator	0 (0.0%)	0 (0.0%)	0 (0.0%)	1.000	0 (0.0%)
Other	0 (0.0%)	1 (1.6%)	0 (0.0%)	.429	1 (0.4%)

In Table 4, LBP treatment history, including LSI(s), LRFA(s), and lumbar surgeries prior to baseline and opioid use at baseline, for BVNA arm aggregate study participants (*N*=247 with a 1-year study visit) are reported by number and percent of participants for the pooled and individual studies.

Abbreviations: BVNA = basivertebral nerve ablation; LRFA = lumbosacral radiofrequency ablation; LSI = lumbosacral spinal injection; SI = sacroiliac.

a BVNA arm cohort with a successful procedure and a 1-year follow-up visit.

b *P* values using Fisher exact test for categorical data and independent sample t-test for continuous data.

c Prior lumbar surgeries such as discectomy or laminectomy were allowed if > 6 months prior to baseline for the INTRACEPT RCT and CLBP Single Arm Cohort Study. Individuals with prior lumbar fusions or total disc replacements were excluded from enrollment in all three trials.

### Aggregate cohort—basivertebral nerve ablation (BVNA) treatment

All vertebral levels exhibiting Modic changes were treated in the 3 individual studies. A blinded, independent interventional radiologist confirmed targeting as well as treatment of all Modic-involved levels. Table 5 reports the vertebral levels treated for each study and the aggregate cohort. The most common vertebral levels for treatment in this cohort were L5 at 97.2%, followed by S1 at 70.9%, L4 at 44.9%, and L3 at 7.3%. There were no significant differences in vertebral levels treated between the 3 study populations included in the aggregate cohort.

**Table 5.** Aggregate cohort vertebral bodies treated.

Vertebral Bodies Treated	SMART BVNA Arm (*N* = 141)^a^	INTRACEPT BVNA Arm (*N* = 61)^a^	CLBP Single Arm Cohort BVNA Arm (*N* = 45)^a^	*P* value^b^	BVNA Aggregate Cohort (*N* = 247)^a^
L3, n (%)	9 (6.4%)	6 (9.8%)	3 (6.7%)	.628	18 (7.3%)
L4, n (%)	59 (41.8%)	33 (54.1%)	19 (42.2%)	.262	111 (44.9%)
L5, n (%)	136 (96.5%)	60 (98.4%)	44 (97.8%)	.873	240 (97.2%)
S1, n (%)	105 (74.5%)	38 (62.3%)	32 (71.1%)	.229	175 (70.9%)

In Table 5, vertebral bodies that were treated in the aggregate cohort and individual studies are reported. The most common vertebral treatment levels in this cohort were L5 and S1.

Abbreviations: BVNA = basivertebral nerve ablation; L = lumbar; S = sacral.

a BVNA arm cohort with a successful procedure and a 1-year follow-up visit.

b *P* values using Fisher exact test for categorical data.

### Aggregate BVNA arm One-Year cohort utilization Post-BVNA

In the 247 aggregate BVNA arm participants with a 1-year follow-up visit, statistically significant reductions in conservative treatments, opioid use, and lumbosacral injections were observed post-BVNA treatment. Conservative treatments, including physical therapy, acupuncture, and chiropractic treatments, were initiated in the year prior to baseline in 38.9% of BVNA arm study participants compared to 11.7% initiated in the 1-year post-BVNA; a 27.1% reduction in conservative treatments (*P *<* *.001; 95% CI 19.8% to 34.5%). Thirty-one percent (77/247) of participants were prescribed opioids at baseline compared to 22.7% at one-year post BVNA respectively; a reduction of 8.5% (*P* = .002; 3.1%, 13.9%). Of the 77 participants taking opioids at baseline, a total of 46/77 (59.7%) remained on opioids at 1 year, a reduction of 40.3% (*P* < .001; 95% CI 28.0% to 52.5%).

Eighty-five participants (34.4%) had 158 therapeutic LSIs in the 1-year prior to baseline compared to 29 participants (11.7%) having 53 therapeutic LSIs in the 1-year post BVNA; a reduction of 22.7% of participants (*P* < .001; 95% CI 15.7%, 29.7%) and 66.5% in the number of therapeutic LSIs overall. Of the 53 1-year post BVNA LSIs, only 18 (34%) were adjudicated by the independent CEC as being for the same pain source and treatment level(s) relative to baseline assessment. The remaining 66% of LSIs were adjudicated by the CEC as treatment for another source of LBP such as posterior element pain (facet joint or SI joint pain) or different treatment levels. In the 85 participants that received therapeutic LSIs in the 1 year prior to baseline, 16 (6.5%) participants had therapeutic LSIs in the 1-year post-BVNA, representing a reduction of 81.2% in this sub-cohort (*P *<* *.001; 95% CI 71.7% to 90.7%). The number of therapeutic LSIs was reduced in these 85 participants from a mean of 1.9 to 0.4 injections per participant from baseline to 1-year post-BVNA (*P* < .001).

Compared to 4.9% during the 1-year prior to baseline, only 1.6% (*n* = 4) of the BVNA-treated participants had 7 LRFAs (5 facet joint and 2 SI joint radiofrequency ablation procedures) through 1-year post-BVNA. None of the post-BVNA LRFAs were adjudicated by the independent CEC to be for the same pain source and treatment level as the BVNA. Four (1.6%) of the 247 study participants had lumbosacral surgery within 1-year post-BVNA (2-lumbar fusions, 1-total disc replacement, and 1-laminectomy). Two of the surgeries were adjudicated by the independent CEC as treatment for *a different pain source* (1-fusion for disc collapse with stenosis and 1-laminectomy treating spinal canal narrowing). See Table 6 for comparisons of utilization at 1-year pre- and post-BVNA.

**Table 6.** 1-year utilization pre and post Basivertebral Nerve Ablation (BVNA).

Treatment Type	1 Year Prior to Baseline (*N* = 247)	1 Year Post-BVNA (*N* = 247)	Difference in Proportions Post- vs Pre-BVNA	95% CI	*P* value^a^
Conservative care, n (%)	96 (38.9%)	29 (11.7%)	−27.1%	−19.8, −34.5	**<.001**
Acupuncture	13 (5.3%)	2 (0.8%)	−4.5%	−1.0, −7.9	**.007**
Chiropractic	31 (12.6%)	6 (2.4%)	−10.1%	−5.5, −14.8	**<.001**
Physical therapy	74 (30.0%)	25 (10.1%)	−19.8%	−12.8, −26.8	**<.001**
Opioids at baseline and 1 year post−BVNA, n (%)	77 (31.2%)	56 (22.7%)	−8.5%	−3.1, −13.9	**.002**
Of those taking opioids at baseline, n (%)	77 (100.0%)	46 (59.7%)	−40.3%	−28.0, −52.5	**<.001**
Diagnostic LSIs, n (%)	2 (0.8%)	5 (2.0%)	+1.2%	+3.7, −1.3	.453
Epidural	0 (0.0%)	0 (0.0%)	0.0%	+0.4, −0.4	1.000
Intradiscal	0 (0.0%)	0 (0.0%)	0.0%	+0.4, −0.4	1.000
Facet joint	2 (0.8%)	5 (2.0%)	+1.2%	+3.7, −1.3	.453
Nerve root	0 (0.0%)	0 (0.0%)	0.0%	+0.4, −0.4	1.000
SI joint	0 (0.0%)	0 (0.0%)	0.0%	+0.4, −0.4	1.000
Trigger point	0 (0.0%)	0 (0.0%)	0.0%	+0.4, −0.4	1.000
Other/unknown	0 (0.0%)	0 (0.0%)	0.0%	+0.4, −0.4	1.000
Therapeutic LSIs, n (%)	85 (34.4%)	29 (11.7%)	−22.7%	−15.7, −29.7	**<.001**
Number of LSIs per participant (all participants), mean, median (range)^b^	0.7, 0, (0, 6)	0.2, 0, (0, 9)			**<.001**
Number of LSIs per participant (those with ≥ 1 LSI), mean, median (range)^b^	1.9, 2 (1, 6)	1.8, 1 (1, 9)			.911
Epidural, n (%)	68 (27.5%)	20 (8.1%)	− 19.4%	−12.7, −26.1	**<.001**
Intradiscal, n (%)	3 (1.2%)	1 (0.4%)	−0.8%	+1.2, −2.8	.625
Facet joint, n (%)	23 (9.3%)	7 (2.8%)	−6.5%	−2.4, −10.5	.001
Nerve root, n (%)	4 (1.6%)	3 (1.2%)	−0.4%	+2.1, −2.9	1.000
SI joint, n (%)	0 (0.0%)	1 (0.4%)	+0.4%	+1.6, −0.8	1.000
Trigger point, n (%)	2 (0.8%)	3 (1.2%)	+0.4%	+2.6, −1.8	1.000
Other/unknown, n (%)	1 (0.4%)	0 (0.0%)	−0.4%	+0.8, −1.6	1.000
Participants with LSI(s) 1-year prior to baseline), n (%)	85 (100%)	16 (18.8%)	−81.2%	−71.7, −90.7	**<.001**
Number of LSIs per participant, mean, median, (range)^b^	1.9, 2, (1, 6)	0.4, 0, (0, 9)			**<.001**
Days to first LSI post-BVNA, median, (range)		169, (39, 378)			
LRFA, n (%)	12 (4.9%)	4 (1.6%)	−3.2%	+0.1, −1.5	.057
Medial branch	12 (4.9%)	4 (1.6%)	−3.2%	+0.1, −6.6	.057
Lateral branch	0 (0.0%)	1 (0.4%)	+0.4%	+1.6, −0.8	1.000
Basivertebral nerve	0 (0.0%)	0 (0.0%)	0.0%	+0.4, −0.4	1.000
Lumbosacral surgeries, n (%)^c^	0 (0.0%)	4 (1.6%)	+1.6%	+3.6, −0.4	.125
Discectomy	0 (0.0%)	0 (0.0%)	0.0%	+0.4, −0.4	1.000
Laminectomy/laminotomy	0 (0.0%)	1 (0.4%)	+0.4%	+1.6, −0.8	1.000
Fusion	0 (0.0%)	2 (0.8%)	+0.8%	+2.3, −0.7	.500
Total disc replacement	0 (0.0%)	1 (0.4%)	+0.4%	+1.6, −0.8	1.000
Neurostimulator	0 (0.0%)	0 (0.0%)	0.0%	+0.4, −0.4	1.000
Other	0 (0.0%)	0 (0.0%)	0.0%	+0.4, 0.4	1.000

Table 6 reports LBP treatments, by number and percent of participants, at 1-year pre- and 1-year post-BVNA for the full cohort of 247 BVNA treatment arm participants with a 1-year visit. There were significant reductions in the proportion of participants receiving conservative therapies, opioid medications, and therapeutic lumbosacral spinal injections in the 1-year post-BVNA compared to the 1-year preceding baseline.

Abbreviations: BVNA = basivertebral nerve ablation; CI = confidence interval; LSI = lumbosacral spinal injection; LRFA = lumbosacral radiofrequency ablation; SI = sacroiliac.

a *P* values is calculated using McNemar's test.

b Paired *t*-test used to compare the mean number of LSIs per participant.

c Individuals with prior lumbar fusions or total disc replacements were excluded from the pooled studies.

### Aggregate BVNA arm Long-Term cohort utilization results

In the 205 aggregate BVNA arm participants in the optional long-term follow-up study, statistically significant reductions in opioid use and therapeutic spinal injections were observed at a mean of 5.3 ± 1.33 years (range 3.2 to 7.9 years) post-BVNA treatment. In the long-term cohort, 60/205 participants (29.3%) were taking opioids at baseline compared to 18/205 (8.8%) actively taking opioids (defined as >25% of the monthly prescribed dose) at a mean of 5.3 years, representing a reduction from baseline of 70.0% (*P* < .001; 95% CI 56.7%, 83.3%) in this sub-cohort. In the long-term cohort, 60/205 participants (29.3%) were taking opioids at baseline compared to 23/205 (11.2%) taking opioids at a mean of 5.3 years, representing a reduction from baseline of 61.7% (*P* < .001; 95% CI 47.7%, 75.6%) in this sub-cohort.

Fifty-four percent (110/205) of participants in the long-term cohort received 274 therapeutic LSIs (average of 2.5 injections per participant who received injections; range 1 to 13) in the 5years prior to baseline. This proportion was reduced to 20.0% (41/205) of participants who received a total of 95 therapeutic LSIs (average of 2.3 injections per participant who received injections; range 1 to 10) in the mean of 5.3 years following BVNA, a reduction of 33.7% (*P* < .001 and 95% CI 24.8% to 42.5%) and a reduction of 65.3% in the number of injections, in this sub-cohort. Of these, 11/205 (4.3%) of participants had 33 spinal injections that were adjudicated by the independent CEC to be for the same vertebrogenic pain source and treatment level.

Seventeen (8.3%) BVNA-treated participants in the long-term follow-up study received 37 LRFAs (34-lumbar facet joint, 2-SI joint, and 1-basivertebral nerve radiofrequency ablation procedures) over a mean of 5.3 years. Of these, 7/37 (19.0%) of the LRFAs (6-facet and 1-basivertebral nerve) performed in two participants were conservatively adjudicated (based on a lack of response to the additional treatment) as for the same vertebrogenic pain source and treatment level as the BVNA by the independent CEC. The remaining 81% of LRFAs were adjudicated as treatment for separate posterior element pain sources by the CEC (based on response to the additional treatment).

Fourteen participants (6.8%) had a spinal fusion through the mean follow-up of 5.3 years post-BVNA (including 2 participants that had a fusion after their 1-year visit but did not have a long-term visit the rate would be 6.5% in the full cohort of 247). Of these, 13/16 (81.2%) were adjudicated by the independent CEC as treatment for the same pain source and vertebral levels. The remaining three lumbosacral spine fusions were for 1-motor vehicle accident trauma, 1- disc collapse, and 1-for treatment of spinal stenosis at a different level than BVNA. Eight of the 16 (50%) lumbosacral spinal fusions in the long-term cohort were performed at a single institution (that treated approximately 9% of the study participants). The remaining lumbosacral spinal fusions were performed at 8 different study sites.

Four discectomies were reported in 3 (1.5%) long-term participants following BVNA. All 4 were adjudicated from other pain sources involving new symptoms from disc herniation, with 2 occurring at a different treatment level than the BVNA. Three participants had a laminectomy following BVNA; all 3 were for other pain sources (1 with identified trauma resulting in new disc herniation and 2 for radiculopathy due to stenosis). One participant (0.5%) had a total disc replacement following BVNA (without response) that was adjudicated as for the same pain source and treatment level by the CEC. One participant had a neurostimulator implanted post-lumbosacral spinal fusion that was conservatively adjudicated by the CEC as DDD anterior element pain source (with treatment failure for BVNA as it involved the same vertebral levels as BVNA); this participant did not respond to BVNA, fusion, or spinal cord stimulation. Comparisons of long-term utilization pre- and post-BVNA are shown in Table 7.

**Table 7.** Long-term utilization post Basivertebral Nerve Ablation (BVNA).

Treatment Type^a^	5 Years Prior to Baseline (N=205)	Latest Follow-Up Post-BVNA (N=205)	Difference in Proportions Post- vs Pre-BVNA	95% CI	*P* value^b^
Opioids at baseline and latest follow-up post-BVNA, n (%)	60 (29.3%)	27 (13.2%)	−16.1%	−9.9, −22.3	**<.001**
Of those taking opioids at baseline	60 (100.0%)	23 (38.3%)	−61.7%	−47.7, −75.6	**<.001**
Actively taking opioids^c^	60 (100.0%)	18 (30.0%)	−70.0%	−56.7, −83.3	**<.001**
Diagnostic LSIs, n (%)	3 (1.5%)	8 (3.9%)	+2.4%	+6.1, −1.2	.227
Epidural	0 (0.0%)	0 (0.0%)	0.0%	+0.5, −0.5	1.000
Intradiscal	0 (0.0%)	0 (0.0%)	0.0%	+0.5, −0.5	1.000
Facet joint	3 (1.5%)	8 (3.9%)	+2.4%	+6.1, −1.2	.227
Nerve root	0 (0.0%)	0 (0.0%)	0.0%	+0.5, −0.5	1.000
SI joint	0 (0.0%)	0 (0.0%)	0.0%	+0.5, −0.5	1.000
Trigger point	0 (0.0%)	0 (0.0%)	0.0%	+0.5, −0.5	1.000
Other/unknown	0 (0.0%)	0 (0.0%)	0.0%	+0.5, −0.5	1.000
Therapeutic LSIs, n (%)	110 (53.7%)	41 (20.0%)	−33.7%	−24.8, −42.5	**<.001**
Number of LSIs per participant (all participants), mean, median (range)^d^	1.3, 1 (0, 13)	0.5, 0 (0, 10)			**<.001**
Number of LSIs per participant (those with ≥ 1 LSI), mean, median (range)^d^	2.4, 2 (1, 13)	2.3, 2 (1, 10)			.634
Epidural, n (%)	94 (45.9%)	26 (12.7%)	−33.2%	−24.7, −41.6	**<.001**
Intradiscal, n (%)	6 (2.9%)	3 (1.5%)	−1.5%	+1.5, −4.5	.453
Facet joint, n (%)	27 (13.2%)	9 (4.4%)	−8.8%	−3.2, −14.4	**.001**
Nerve root, n (%)	5 (2.4%)	4 (2.0)	−0.5%	+2.5, −3.5	1.000
SI joint, n (%)	1 (0.5%)	5 (2.4%)	+2.0%	+4.8, −0.9	.219
Trigger point, n (%)	3 (1.5%)	4 (2.0%)	+0.5%	+3.5, −2.5	1.000
Other/unknown, n (%)	3 (1.5%)	1 (0.5%)	−1.0%	+1.4, −3.4	.625
Participants with LSI(s) 5 years prior to baseline, n (%)	110 (100%)	26 (23.6%)	−76.4%	−67.5, −85.2	**<.001**
Number of LSIs per participant, mean, median (range)^d^	2.5, 2, (1, 13)	0.7, 0, (0, 10)			**<.001**
Days to first LSI post-BVNA, median, (range)		365, (40, 1977)			
LRFA, n (%)	17 (8.3%)	17 (8.3%)	0.0%	+5.4, −5.4	1.000
Facet joint	17 (8.3%)	16 (7.8%)	−0.5%	+4.8, −5.8	1.000
SI joint	0 (0.0%)	1 (0.5%)	+0.5%	+1.9, −1.0	1.000
Basivertebral nerve	0 (0.0%)	1 (0.5%)	+0.5%	+1.9, −1.0	1.000
Lumbosacral surgeries, n (%)^e^	8 (3.9%)	19 (9.3%)	+5.4%	+10.8, −0.0	**.052**
Discectomy/microdiscectomy	4 (2.0%)	3 (1.5%)	−0.5%	+2.5, −3.5	1.000
Laminectomy/laminotomy	4 (2.0%)	3 (1.5%)	−0.5%	+2.5, −3.5	1.000
Fusion	0 (0.0%)	14 (6.8%)	+6.8%	+10.8, +2.9	**<.001**
Total disc replacement	0 (0.0%)	1 (0.5%)	+0.5%	+1.9, −1.0	1.000
Neurostimulator	0 (0.0%)	1 (0.5%)	+0.5%	+1.9, −1.0	1.000
Other	1 (0.5%)	0 (0.0%)	−0.5%	+1.0, −1.9	1.000

Table 7 reports LBP treatments, by number and percent of participants, at 5-years pre- and latest follow-up post-BVNA for the long-term cohort of 205 BVNA treatment arm participants with at least one long-term visit. There were significant reductions in the proportion of participants taking opioid medications and receiving therapeutic lumbosacral spinal injections in the mean of 5.3 years post-BVNA compared to the 5-years preceding baseline.

Abbreviations: BVNA = basivertebral nerve ablation; CI = confidence interval; LSI = lumbosacral spinal injection; LRFA = lumbosacral radiofrequency ablation; SI = sacroiliac.

a Conservative care data only collected through 24 months.

b *P* value is calculated using McNemar's test.

c Actively taking opioids is defined as 25% or more of total prescribe dosage in the 30 days prior to study visit.

d Paired *t*-test used to compare the mean number of LSIs per participant.

e Individuals with prior lumbar fusions or total disc replacements were excluded from the pooled studies.

### Utilization survival curves

**Figure 2.**
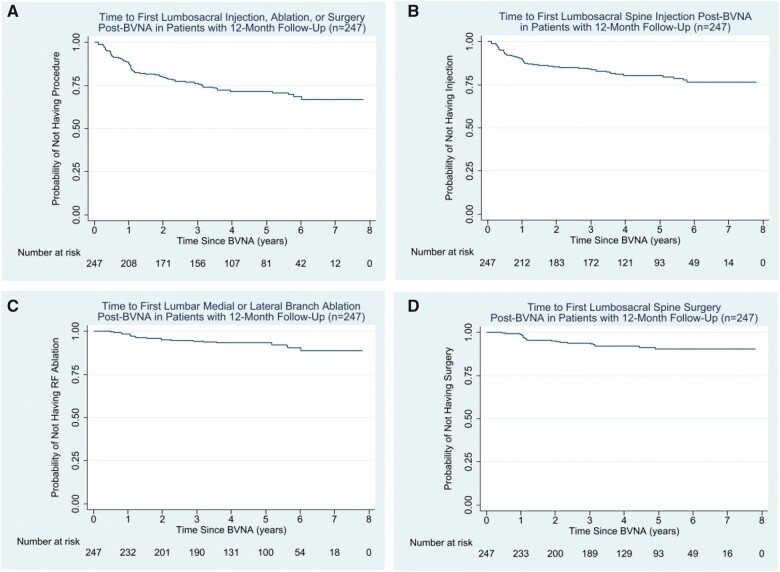
(**A**) Depicts the time post-BVNA to first additional lumbosacral therapeutic procedure [spinal injection (LSI), lumbosacral radiofrequency ablation (LRFA), and/or lumbosacral spine surgery] in the full cohort of *N* = 247 participants with a 1-year follow-up. (**B)** Depicts the time post-BVNA to first lumbosacral spinal injection (LSI) in the full cohort of *N* = 247 participants with a 1-year follow-up. (**C**). Depicts the time post-BVNA to first lumbosacral radiofrequency ablation (LRFA) in the full cohort of *N* = 247 participants with a 1-year follow-up. (**D**) Depicts the time post-BVNA to first lumbosacral spinal surgery in the full cohort of *N* = 247 participants with a 1-year follow-up.

Kaplan-Meier analyses were used to determine the average time to first LBP-related treatment procedure after BVNA in the 247 BVNA arm participants with a one-year follow-up. The Kaplan-Meier survival curves depict the probability of not having a treatment event (therapeutic LSI, LRFA, or lumbosacral spine surgery) over time after BVNA. The x-axis represents years since BVNA; and the y-axis represents the proportion of participants who have not had a procedure at each timepoint. For example, in Figure 2A, at 3 years post-BVNA, 75% of participants had not received any therapeutic LSIs, LRFAs, or lumbosacral surgery. The number at risk at the bottom of each figure is a count of the number of participants with follow-up data at the specific timepoint and thus who are still at risk of having a treatment procedure post-BVNA. Participants are removed from this “risk pool” upon experiencing a treatment event (therapeutic LSI, LRFA, or lumbosacral surgery) or if they left the study before experiencing an event (censored). For example, in Figure 2A at year six there were 42 participants remaining who had not undergone a treatment event and who had not yet left the study. See Figure 2A–E.

## Discussion

This report provides pooled healthcare utilization results for BVNA-treated participants from three prospective clinical trials that included individuals with severe pain and significant LBP-related disability of presumed vertebrogenic origin. We report statistically significant and clinically meaningful reductions in LBP-related healthcare utilization from pre-treatment baseline to both one-year and a mean of 5.3 years post-BVNA treatment. In participants on opioids at baseline, opioid use was reduced by 40.3% at 1 year and 61.7% at 5 years. The number of therapeutic lumbosacral spine injections were reduced by 66.5% at one-year and 65.3% at a mean of 5.3 years following BVNA. The reduction in opioid use is particularly poignant given copious published literature indicating that both patients and their prescribing providers are reticent to wean or discontinue opioids due to fears of adverse events, including withdrawal and relapse of symptoms.^27^^,^^28^ Indeed, the literature is mixed regarding the ability and magnitude of various invasive procedures to facilitate reduction or cessation of opioid use in individuals with chronic LBP.^29^

Rates of LRFA and surgeries were low during a mean of 5.3 years following BVNA in this aggregate analysis. In total, during this time only 17 (6.9%) participants had one or more LRFA procedures and 20 (8.1%) had 25 lumbosacral surgeries. Of these, 2 participants (0.8%) had 7 RFA procedures and 14 participants (5.7%) had 15 lumbosacral surgeries (13-fusion, 1-TDR, 1-spinal cord stimulator implant) that were adjudicated by an independent CEC or orthopedic surgeons to be treatment for the same pain source and vertebral levels. Notably, 1 study site was an outlier, accounting for 50% of the lumbosacral fusion surgeries performed across all study sites, compared to the remaining eight fusions being spread across 8 different study sites. If the outlier site is removed, the rate of lumbosacral surgeries is 3.2% at a mean of 5.3 years following BVNA.

The low rate of pain interventions and spine surgeries following BVNA is notable in a population with severe CLBP (mean baseline VAS = 6.8), where at the time of baseline assessment 66.8% of participants reported experiencing low back pain for ≥ 5 years, 31.6% were taking opioids, 33.6% reported clinically relevant anxiety/depression, and their physical function was severely impacted (mean baseline ODI = 44.4). Reduced healthcare utilization is also particularly compelling as observed more than 5 years post-BVNA given that this population included 79.0% of participants with advanced degenerative disc disease (DDD) (Pfirrmann Grades 4 and 5) at baseline. It is important to highlight that further disc height loss and other degeneration is expected by natural history in a proportion of individuals with advanced DDD, which may lead to a new disc herniation, worsening spinal stenosis, or posterior element loading with pain. This appears to be the case in a minority of participants in the present study with less than 10% receiving lumbosacral RFA (6.9%) or lumbosacral surgery (8.1%) during a mean 5-year follow-up. When considering all of the additional pain interventions and surgeries over the mean 5.3-year follow-up post-BVNA, 64.5% of these procedures were performed for treatment of other pain sources.

Candidates for BVNA are likely to fall into the moderate and high-impact pain categories as defined by the US Department of Health and Human Services,^30^ with high pain intensity and variable impact on activity from low to substantial restrictions. Herman et al. found that such individuals with moderate and high impact chronic LBP had annual per person reported healthcare expenditures of $9657 and $14 661, respectively.^31^ Much of the increased costs were deemed secondary to increased rates of interventional pain procedures, including injections and radiofrequency ablation procedures, though opioid usage was also demonstrated to increase with impact level. A separate study conducted by Spears et al. evaluating healthcare utilization in 50,000 non-surgically managed patients with 12 months of persistent LBP found that in the subsequent year, median cost nearly doubled from $3732 baseline cost to $6,590, with outpatient services, including interventional pain procedures, accounting for the majority of total costs.^32^

Pain intervention and surgery rates following BVNA in our study are low compared to the rates observed in studies of hundreds-of-thousands of U.S. patients with chronic low back pain, using private insurance care claims data (MarketScan^®^ Commercial Claims and Encounters Database) in which the rate of medial branch RFA was 26.7%^33^ during the first year after a facet joint intervention and the rate of spinal surgery after epidural steroid injection was 16.9% at 1 year and 26.1% at 5 years.^34^

Spinal fusion for axial LBP has resulted in scrutiny given unclear benefit and substantial costs. Martin et al. reported that, despite a lack of evidence for lumbar fusion surgery in treatment of CLBP in the absence of instability, rates of elective lumbar fusion increased 62.3% from 2004 to 2015.^35^ Mirza et al. conducted a longitudinal study of 495 participants with isolated DDD and CLBP with axial-predominant symptoms and no evidence of instability across 5 study sites.^36^ The full inclusion and exclusion criteria were remarkably similar to the present study (with the exception of Type 1 or 2 Modic changes required by the present study). Inevitably, a subset of these patients had Type 1 or 2 Modic changes associated with degenerative disc disease (DDD), but this proportion was not reported. The authors observed a 17% rate of surgery within 6 months of enrollment. By a mean of 2.4 months post-enrollment, 14% had a lumbar spinal fusion, 2% had TDR, and 2% had a laminectomy/discectomy.^36^ In comparison, the rates of spinal surgery reported in the present study are much lower—8.1% in total including a 6.5% rate of fusion surgery during a mean of 5.3 years post-BVNA. Thus, fusion rates post-BVNA are approximately 50% less than the published rates for patients with chronic axial LBP and DDD. This equates to a potential savings of 7.5 fusions per 100 patients with DDD.

With regard to the cost of care in patients post-spinal fusion, Mina et al. published longitudinal data on 1422 lumbar spinal fusion patients from Cigna’s national claims database, reporting that 87% of patients continued to receive medical and/or pharmaceutical treatment for LBP at an additional cost of $11.2 million ($12 283 per patient) in the two years following their fusion surgery.^37^ The population for this study was young, with 43% under 50 years of age, similar to our study population (mean of 47.3 years). Additional single or multi-level fusions were performed in 13.6% of the study population in the 2 years post-fusion, and 25% had additional pain interventions including epidural steroid injections, facet injections, SI joint injections, trigger point injections, and RFA. More than 62% of patients continued chronic opioid use beyond one-year post-fusion. Of patients taking opioids at baseline, a surprising 95% continued opioids at two years post-fusion. Similarly, a recent review of opioid use following lumbar spinal fusion conducted by Deyo et al. found that more patients received long-term opioids following surgery than were prescribed preoperatively.^38^ That said, the present study’s population was more homogenous than that analyzed by Mino et al. and Deyo et al. suggesting caution in making a direct comparison. Still, the low rate of post-BVNA surgery and interventions, and reduction in opioid use are strong indicators of the value of BVNA.

Combined, the findings in this study indicate a substantial reduction in healthcare utilization for CLBP during a mean of 5.3 years post-BVNA for a specific subgroup of patients with CLBP identified by clinical and radiographic features to be vertebrogenic pain with associated functional disability. All participants in this aggregate analysis of three clinical trials were diagnosed with presumed vertebrogenic pain; however, enrollment was not exclusive of other spinal pathologies. Within this cohort, 79.0% had advanced DDD (Pfirrmann grade IV/V) and 67.2% of participants had foraminal, subarticular zone and/or central canal stenosis without radicular pain/claudication; 7.3% had olisthesis < 2 mm, and 19.4% had disc protrusion less than 5 mm. Eight (8.1%) percent of participants had prior LRFA, and 5.3% had prior lumbosacral surgeries. The significant reduction in utilization of opioids and spine interventions, and low rates of surgery over the 5.3-year period in this study suggests that BVNA treatment is beneficial even in the presence of other radiographic spinal pathology.

Strengths of this aggregate utilization analysis include homogenous cohorts using similar inclusion/exclusion criteria, study timeframes, and clinical endpoints. While there were significant differences in age and the Beck’s Depression Index at baseline between study populations, these were demonstrated in prior predictive analyses to be weak predictors of treatment failure^26^ thus potentially leading to more utilization post BVNA. The attrition rate at 1 year and 5.3 years was low with retention rates of 95% and 79%, respectively. In addition, there were no protocol-driven or required treatment algorithms post-BVNA that would bias these utilization results. In fact, decisions about all treatments after BVNA were made in a manner that is consistent with typical clinical practice using a joint decision-making process between participants and their treating physicians based on each participants’ unique symptoms.

Limitations include the fact that all data were derived from open-label, industry-sponsored studies. In addition, there was no long-term comparator group for utilization in the non-surgical care arm due to a cross-over design in each of the two RCTs. However, average utilization for a non-surgical care arm would be expected to follow published reports for patients with DDD followed longitudinally where fusion rates are 14% in a mean of 2.4 months.^36^ Utilization may be underreported pre-baseline as historical medical records may not have been comprehensive. However, this would also limit the ability to demonstrate reductions in utilization post BVNA. Likewise, with a retention rate of 79% at 5-years post BVNA ablation may have been underrepresented. However, in those who did not participate in the long-term study (*n* = 54) there were 9 (16.6%) with LSIs, 2 (3.7%) with LRFAs, and 2 (3.7%) with spinal fusions that were reported prior to exiting the study. Therefore, it is reasonable to deduce that utilization patterns seen in the long-term sub-study would be applicable more broadly.

## Conclusions

In this aggregate analysis of healthcare utilization post BVNA, the use of non-invasive conservative care, opioids, lumbosacral spinal injections, and lumbosacral RFA were significantly reduced through five years compared to utilization rates at baseline. At a mean follow-up of 5.3 years, the rate of fusion surgery was low (6.5%) and less than half the published rate of 14% at 6 months in patients with chronic LBP and DDD.
